# Dermatological Autoimmune Considerations of Immune Checkpoint Therapy

**DOI:** 10.3390/biomedicines10102339

**Published:** 2022-09-20

**Authors:** Lauren S. Fane, Jimmy T. Efird, Charulata Jindal, Tithi Biswas

**Affiliations:** 1 MD University Program, Case Western Reserve University School of Medicine, Cleveland, OH 44016, USA; 2 VA Cooperative Studies Program Coordinating Center, Boston, MA 02130, USA; 3 Department of Radiation Oncology, Case Western Reserve University School of Medicine, Cleveland, OH 44015, USA; 4 Harvard Medical School, Harvard University, Boston, MA 02115, USA; 5 University Hospitals Cleveland Medical Center, Case Western Reserve University School of Medicine, Cleveland, OH 44015, USA

**Keywords:** cutaneous immune-related adverse events, immune checkpoint inhibitors, racial disparities, radiotherapy, skin autoimmune diseases, steroids

## Abstract

The most common immune-related adverse events (irAEs) involve the skin, and several serve as predictors of response to immune checkpoint inhibitor (ICI) therapy, especially in melanoma. Patients with pre-existing skin autoimmune diseases (ADs) have been excluded from ICI studies for safety concerns, yet recent research has shown that dermatological ADs can be managed without discontinuing ICI therapy. Patients with ADs respond as well or better to ICIs and can be included as candidates in clinical trials. Frequently taken during ICI therapy, steroids impair immunotherapy efficacy in certain anatomical sites of tumors but not others, including the brain. ICI efficacy can be enhanced by radiotherapy without increasing adverse events, as neoadjuvant radiotherapy is thought to sensitize tumors to ICIs. This perspective highlights clinical autoimmune considerations of ICI therapy in melanoma and discusses important areas for future exploration.

## 1. Introduction—Prognostic Value of Immune-Related Adverse Events

Immune-related adverse events (irAEs) are cancer treatment side effects that have evolved with the introduction of immune checkpoint inhibitors (ICIs). Since melanoma was the first cancer approved for ICI therapy and frequently has cutaneous irAEs, these irAEs have been well studied and managed by clinicians. Cutaneous irAEs tend to manifest earlier than those of other organ systems, alerting clinicians of their potential future occurrence [1]. The order in which irAEs develop tends to follow a sequential pattern based on the ICI drug. For example, melanoma patients treated with nivolumab have an onset of dermatologic irAEs at 5 weeks, gastrointestinal at 7 weeks, hepatic at 8 weeks, pulmonary at 9 weeks, endocrine at 10 weeks, and renal toxicity at 15 weeks [2]. IrAEs tend to manifest similarly based on the ICI agent, across various cancer sites. Table 1 summarizes the common irAEs stratified by ICI therapeutic target and cancer site [3,4,5,6].

While irAEs can increase morbidity and decrease quality of life, some are associated with improved tumor response to ICIs. Cutaneous irAEs tend to be low-grade, and certain conditions have been reported as positive prognosticators [2]. Which irAEs are positive or negative predictors remains debated as studies have yielded conflicting results. Vitiligo (skin depigmentation) is well-documented to be a favorable predictor, and multiple pathways have been posited to explain its relationship to ICI mechanisms of action [7]. Nonetheless, a recent study reported a cohort of patients with ICI efficacy associated with lichenoid and psoriasiform irAEs but not the irAEs reported by previous studies (e.g., vitiligo, isolated pruritus, and maculopapular reactions) [8].

**Figure 1:**
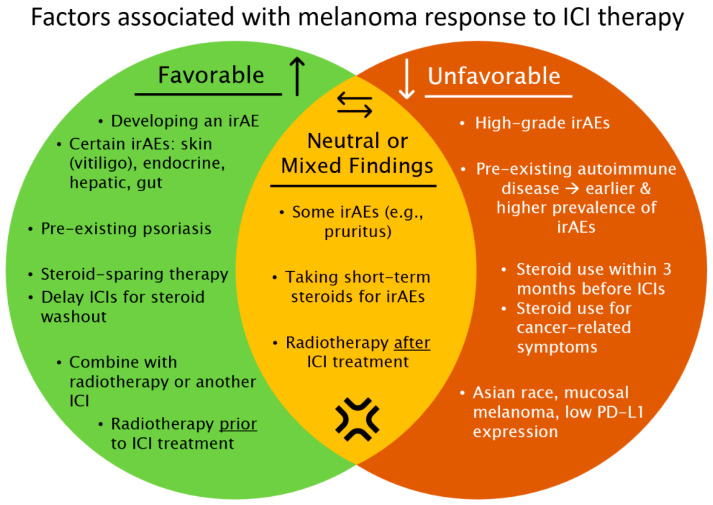
A summary of factors associated favorably and unfavorably with melanoma response to immune checkpoint inhibitor (ICI) therapy. irAE = immune-related adverse event.

With more malignancies and populations undergoing ICI therapy, management of cutaneous irAEs will continue as an important focus for dermatologists and oncologists. Progress in understanding the dermatological autoimmune aspects of ICIs can serve as a basis for investigating other immunogenic cancers and associated irAEs. This perspective provides an update on important areas of focus and future discovery regarding dermatological autoimmune aspects of ICI therapy (Figure 1).

## 2. Pre-Existing Autoimmune Skin Diseases in ICI Cancer Patients

ICI clinical trials initially excluded patients with pre-existing autoimmune diseases (ADs). However, pilot studies have shown that ADs can be managed without discontinuing ICI treatment [9]. As ICIs have become a cornerstone of treating metastatic melanoma, it is important to know whether patients with ADs can safely receive this immunotherapy [10].

A retrospective analysis of 76 patients on ICIs with pre-existing psoriasis found frequent psoriatic exacerbations, but the flares were manageable with conventional treatments [11]. Few patients (7%) required ICI discontinuation. Patients who developed a psoriatic flare had a longer progression-free survival than patients who did not develop a psoriatic flare (39 vs. 9 months). Patients who experienced exacerbations had a tumor response at least comparable with those who did not. Therefore, those with pre-existing psoriasis may be considered as candidates for ICI therapy.

Pre-existing and de novo cases of psoriasis were compared in an ICI study of mostly melanoma and lung cancer patients. Seventy percent (70%) of psoriasis cases developed during ICI therapy. Participants with pre-existing psoriasis experienced deterioration of the disease earlier than de novo cases [12]. This cohort had a high ICI response rate, suggesting psoriasis is a positive predictor. The authors recommend strict dermatologic surveillance of psoriasis cases and management with agents that do not interfere with ICI therapy, without discontinuing the cancer treatment.

Of 119 melanoma patients with ADs in a multicentric study, 8 patients had pre-existing psoriasis, of which 3 required immunosuppression during ICI therapy [13]. Although frequent AD exacerbations were induced by anti-PD-1 therapy, they were often mild and easily managed, did not require ICI discontinuation, and were associated with higher clinical response rates. This cohort had higher than expected response rates—33% in those with ADs and 40% in those with irAEs. In comparison, similar anti-PD-1 clinical trials without AD patients had response rates of 21–32%. This suggests that patients with ADs may be more likely than those without to benefit from anti-PD-1 therapy, even though they have higher prevalence of adverse prognostic factors. For patients with ADs considering ICIs, it is important to evaluate whether the benefits outweigh the disadvantages.

Similar results were observed in a study of a combination ICI treatment (anti-PD-1 and ipilimumab). In 55 melanoma patients with ADs, 6 patients had pre-existing psoriasis, of which half experienced a flare [14]. Immunosuppression was associated with a higher risk of a flare occuring, and the overall response rate (55%) was not decreased. Combination therapy in this cohort had comparable efficacy with previous trials of patients without ADs.

These findings increase confidence in offering ICIs to cancer patients with ADs. Additionally, examining the clinical course of dermatological ADs throughout ICI treatment can reveal aspects of their pathogenesis.

## 3. Immunosuppressive Steroid Use during ICI Therapy

Many irAEs and brain metastases common to advanced melanoma are treated with systemic steroids. However, steroids have immunosuppressive properties that interfere with ICI efficacy. Steroids weaken the immune system through various mechanisms, from inhibiting acute inflammation to immunomodulatory effects [15]. Most clinical trials of ICIs have excluded patients taking immunosuppressive agents. Consequently, data is limited on the impact of taking steroids on ICI outcomes, which occurs in the real world. Steroids are widely used in cancer patients, ranging from supportive, symptomatic control for brain metastases to curative treatment for leukemia. Specifically, glucocorticoids are used in combination chemotherapy for hematologic malignancies as they induce apoptosis in these cells [16].

Managing irAEs with steroids does not impact overall survival (OS), according to retrospective studies of melanoma and non-small cell lung cancer [17]. In contrast, patients taking steroids for reasons other than irAEs (e.g., cancer-related symptoms or reducing edema around brain metastases) are at increased risk of death and tumor progression. Confounding factors may contribute to the poor outcomes of patients taking steroids for cancer-related symptoms. Since these symptoms tend to be present before ICI initiation, these patients may have longer steroid exposure. Also, steroids are often prescribed at higher doses for palliation of cancer-related symptoms. Clinicians should exercise caution in administering steroids and may want to consider steroid-sparing alternatives or tapering steroid use prior to immunotherapy. Many clinical trials did not record steroid indication, type, dose, or duration, which limited analyses of timing and intensity of exposure.

A recent study was able to evaluate how the timing of steroid exposure effects ICI efficacy using the SEER-Medicare linked database [18]. Patients with steroid exposure within 3 months before ICI therapy had higher all-cause mortality for up to 6 months after ICIs. Specifically, steroid exposure ≤1 month and >1–3 months before ICI therapy is associated with higher mortality by 126% and 51%, respectively. Accordingly, clinicians may opt for steroid-sparing therapies or delay ICIs for 3 months to allow for steroid elimination.

Brain metastases develop in over 50% of patients with metastatic melanoma and have a dismal prognosis [19]. The steroid dexamethasone is commonly administered palliatively to reduce immune-mediated edema around brain tumors. Since the central nervous system (CNS) is an immune-privileged organ, CNS tumors are more likely to escape immune system detection [20]. Steroids have been shown to exacerbate the risk and their use is contraindicated for ICI treatment of CNS tumors [21].

On the other hand, corticosteroids may differentially impact immunotherapy efficacy based on the anatomical site of the tumor [22,23]. Dexamethasone did not attenuate anti-PD-1-mediated immune responses against CNS tumors but did so for tumors in the periphery. This calls for development of specialized guidelines for patients with CNS tumors regarding steroid use that does not limit anti-PD-1 efficacy.

## 4. ICI Combination Treatments Can Increase Efficacy without Compromising Safety

Given the frequency of irAEs during ICI treatment, the safety profile of ICIs combined with other treatment modalities is important to consider. Combinations of an ICI with another ICI, radiotherapy (RT), or targeted cancer drugs have conferred superior tumor control for metastatic melanoma to the brain [24,25,26].

Comparing ICI + radiotherapy (RT), ICI alone, and RT alone in treating melanoma brain metastasis revealed better survival outcomes in ICI + RT [24]. Grade ≥ 3 neurologic adverse events and radiation necrosis were not significantly increased in ICI + RT than the two monotherapies. Adding RT appears to improve overall survival and has a comparable toxicity profile to ICI alone.

A separate meta-analysis compared ICI combination therapy, ICI + RT, and ICI alone for melanoma brain metastasis. The ICI combination therapy consisted of the PD-1 inhibitor nivolumab and the CTLA-4 inhibitor ipilimumab. Both combination therapies had better local efficacy than ICI monotherapy [25]. Intracranial disease control rate (DCR) was highest in ICI + RT (85%). DCR is the proportion of participants who have a complete response (CR), partial response, or stable disease (i.e., neither tumor regression nor growth). Intracranial complete response (CR) rate was highest in ICI combination therapy (23%). ICI combination therapy had a higher grade 3 and 4 adverse event rate (60%) than ICI + RT (4%) and ICI monotherapy (11%). These results illustrate the improved efficacy and comparable safety of ICI + RT for metastatic melanoma to the brain.

Even with the efficacy of the combined anti-PD-1 and anti-CTLA-4 therapies, resistance is common. Some melanoma patients with high PD-L1 expression do not respond to anti-CTLA-4 + RT. PD-L1 has been found in mouse models to allow tumor cells to escape anti-CTLA4 therapy, and the addition of anti-PD-L1 therapy promoted response [26]. CTLA-4 antibodies inhibit regulatory T cells (Tregs) which promote exhaustion and increase the CD8/Treg ratio. A high CD8/Treg ratio is a favorable prognostic, as CD8+ T cells can kill tumor cells, while Treg cells suppress immune responses. Adding PD-L1 therapy synergistically reversed T cell exhaustion and prevented depression of the CD8/Treg ratio. These findings suggest the potential for combination ICI therapies to treat cancer resistant to current regimens.

The sequence of radiotherapy (RT) and ICI treatment affects clinical outcomes. Treating melanoma brain metastases with RT before ICI conferred superior survival than the opposite sequence [27]. RT sensitizes tumors to ICIs by inducing non-repairable DNA strand breaks, releasing tumor cell contents/antigens, increasing the blood-brain barrier’s permeability, and activating immune cells to attack tumor cells outside the irradiated zone (abscopal effect) [25,28,29]. Combining RT with ICI treatment improves immunotherapy response without compromising safety.

## 5. Race and Melanoma Subtype May Influence ICI Response

The expression of immunomodulatory molecules on tumors have been reported to differ by race and may explain varying sensitivity to ICIs. While there is a paucity of racial disparities research on ICI efficacy, disparities exist between Caucasian and Asian populations that are attributable to melanoma subtype. Initial melanoma ICI clinical trials included few Asian patients. Additionally, there were a lack of mucosal or acral melanoma types which constitute a higher proportion of melanoma cases among Asians (60%) [30,31]. Compared with cutaneous melanoma, mucosal melanoma tends to be diagnosed at advanced stages, since it arises on less visible mucosal membranes inside the body [32,33]. Research conducted in China and Japan have shown differences from Caucasian patients in ICI efficacy and irAEs.

The mucosal type of melanoma is less likely to be PD-L1 positive than the cutaneous types [34,35,36]. Asian patients with melanoma were found to have lower levels of certain ICI therapeutic targets (PD-L1, CTLA-4, and IDO-1) than Caucasian patients in an analysis of RNA sequencing expression profiles [37]. Since the mucosal type and Asian race are associated with lower levels of PD-L1, it follows that Asian melanoma patients would be less responsive to therapies that block the receptor PD-1. Japanese patients had a lower objective response rate (ORR) (24%) for cutaneous melanoma treated with pembrolizumab (anti-PD-1) than non-Japanese patients (33–34%) in the KEYNOTE trials [38]. Similar findings were observed for nivolumab, another anti-PD-1 therapy [34]. Clinicians may want to consider alternative therapies for such patients. For example, a case series of 3 patients with melanoma refractory to nivolumab (anti-PD-1) responded to ipilimumab (anti-CTLA-4) combined with radiotherapy [39].

More robust ethnic disparities research in ICI efficacy is necessary, as findings have had discrepancies. A retrospective study observed that the ORR in Chinese patients to PD-1 inhibitor pembrolizumab was comparable with that of clinical trials in Western countries [40].

The safety profile of ICIs is generally similar between Asian and Caucasian melanoma patients, with a few differences [41]. Chinese patients on pembrolizumab (anti-PD-1) have a higher incidence of liver function damage and a lower incidence of diarrhea. In contrast to previous research, studies in Asian patients found the prognostic value of irAEs to be severity-dependent [40,42]. Compared with Asian patients with no irAEs, those with grade 1 and 2 irAEs had higher ORR and DCR, while those with grade 3 and 4 irAEs had no difference.

The association between Asian ethnicity and mucosal melanoma with lower clinical efficacy for anti-PD-1 therapy are intriguing. Nonetheless, divergent results regarding ethnicity or melanoma subtype will need to be reconciled prior to their incorporation into clinical recommendations.

## 6. Conclusions

Given the attention on melanoma and cutaneous irAEs in the field of ICIs, dermatologists have played an important role in their clinical management and research. Although patients on ICIs with pre-existing autoimmune diseases (ADs) develop irAEs earlier and more frequently, ADs are associated with higher ICI response rates. Most dermatological AD cases can be managed without discontinuing ICI treatment. Therefore, cancer patients with skin ADs need not be excluded as candidates for this immunotherapy.

Steroids are best administered conservatively in managing irAEs and brain metastases during ICI treatment. Methods to safely direct steroid use are under study, including the influence of dosing, delaying ICI therapy for steroid elimination, and using alternative therapies. Further analysis will help confirm if steroid immunosuppression differs sufficiently by the anatomic site to warrant specialized steroid use guidelines.

Combining ICI therapy with other cancer treatments does not compromise their safety profile while improving clinical response. Also, of interest is exploring the molecular biology of how radiotherapy sensitizes cancers to ICIs when applied alone or in tandem with adjuvant therapy.

Furthermore, racial disparities research in ICI response of melanoma patients has identified differences in expression of therapeutic targets between Asian and Caucasian patients. Disparities at the molecular level merit additional investigation to determine how they can be incorporated into clinical decision-making regarding ICI use.

## Figures and Tables

**Table 1:** Overview of frequent irAEs by ICI therapeutic target and cancer site.

ICI Target Receptor	Cancer Site	Common irAEs (Immune-Related Adverse Events)
**Anti-PD-1** Nivolumab Pembrolizumab **Anti-PD-L1** Atezolizumab Avelumab Durvalumab	Melanoma	Dermatologic: vitiligo *, pruritus, maculopapular rash Hepatic: elevated AST/ALT, hepatitis Endocrine: hypothyroidism, hypophysitis GI (gastrointestinal): diarrhea, colitis Pulmonary: pneumonitis
	Non-small cell lung cancer (NSCLC)	Endocrine: thyroiditis *, hypo-/hyper-thyroidism Dermatologic: rash GI: diarrhea Pulmonary: pneumonitis
	Renal cell carcinoma	Dermatologic GI: diarrhea, colitis Endocrine
	Urothelial carcinoma	GI Dermatologic
	Gastrointestinal cancer	Musculoskeletal (MSK) Dermatologic Endocrine GI
	Head & neck cancer	Dermatologic MSK Endocrine
**Anti-CTLA-4** Ipilimumab	Melanoma	GI Dermatological Musculoskeletal Endocrine

* This irAE is reported to be a positive prognostic factor for ICI response.

## Data Availability

Not applicable.
